# Supplementary material for: How Do Hunters Hunt Wild Boar? Survey on Wild Boar Hunting Methods in the Federal State of Lower Saxony
Source: Animals (Basel). 2021 Sep 10;11(9):2658. doi: 10.3390/ani11092658 (PMC8468578; doi:10.3390/ani11092658)
Supplement: Supplementary file 1 [file animals-11-02658-s001.zip › animals-1326166-supplementary.pdf]

animals

MDPI

Supplemental Material

How do hunters hunt wild boar?

Inquiries on hunting methods in Lower Saxony, northern Germany, as a basic for management considerations

Oliver Keuling[1]*, Egbert Strauß[1,2], Ursula Siebert[1]

[1] 1Institute for Terrestrial and Aquatic Wildlife Research, University of Veterinary Medicine Hannover, Bischofsholer Damm 15, 30173 Hannover, Germany, oliver.keuling@tiho-hannover.de / ursula.siebert@tiho-hannover.de

[2] Hunting Association of Lower Saxony (Landesjägerschaft Niedersachsen e.V.), Schopenhauerstr. 21, 30625 Hannover, Germany, egbert.strauss@tiho-hannover.de

* Correspondence: oliver.keuling@tiho-hannover.de; Tel.: +49-511-856-7396

animals MDPI

Supplemental Material S1

Definition of the German hunting regime

The German hunting regime is regulated in the hunting act (Bundesjagdgesetz BJagdG) [92]: Section II, §4-§10a.

In the Federal Republic of Germany (and thus, in the Federal State of Lower Saxony) hunting regime is organised in hunting grounds (see Tab. SM1). The shooting right (concession for hunting at all) is by law strictly connected to the land ownership. This concession may be leased to legal hunters. Hunting (management) on land owned by private landowners (persons or companies) usually is conducted in own property (EJB) or leased to other private hunters (EJB and GJB, see Tab. S1). Land in property of the Federal Republic of Germany, the 16 Federal States, or of church, municipalities etc. is also regulated under the definitions in Table S1. Larger state owned areas are EJB, smaller areas than 75 ha are belonging to common hunting grounds (GJB). State owned hunting grounds (states forestry, church, municipalities…) are usually under supervision of forestry officers, who are by definition and education professional hunters (PH). Owners or tenants of the private hunting grounds hunt in personal responsibility with a compulsory duty for wildlife management (given by hunting law: "Pflicht zur Hege"; duty for population regulation, custody and conservation, BMEL 2015). Game species populations must not increase above a certain density compatible with agricultural or social interests. In Germany, no additional management of game species by official game keepers (like the "Wildhut" in Switzerland) is conducted.

Table S1: Definition and minimum sizes of hunting grounds

"Eigenjagdbezirk" EJB<br>hunting ground in one selves property	min. 75 ha<br>consecutive area owned by one single person, or e.g. corporation, association, company, or governmental/administrative institution<br>no upper limit
"Gemeinschaftlicher Jagdbezirk" GJB<br>common hunting ground	min. 150ha<br>larger GJB may be divided into smaller subdivisions with at least 250 ha each<br>several landowners, belonging to one village district<br>no upper limit

It is important to distinguish between private hunters (own responsibility for hunting ground and game), professional hunters (employed professional hunters or forestry officers, responsible for common or private hunting grounds, professionally educated) and some few recreational hunters (using a kind of license hunting system without any responsibilities just for leisure).

Hunting licenses are handed out by authorities solely after passing a high quality exam and may be renewed every three years regardless of the status private or professional hunter.

References SM1

92. BMEL (2015) Bundesjagdgesetz in der Fassung der Bekanntmachung vom 29. September 1976 (BGBl. I S. 2849), das zuletzt durch Artikel 422 der Verordnung vom 31. August 2015 (BGBl. I S. 1474) geändert worden ist. Bundesministerium für Ernährung und Landwirtschaft, Bonn

animals MDPI

Supplemental Material S2

Presentation of Questions

The typical head of the front-page contains data on the hunting ground in every year: corresponding number of hunting ground, local and regional hunting association, municipality, size of hunting ground and different habitat proportions within hunting ground.

Surveys 2004-2008:

Census of wild boar in Lower Saxony

Please help to get an overview on the wild boar population and the hunting conducted:

Number of shot wild boar in total in your hunting ground	N =
Thereof:	
Single hunt (stalking, hiding)	N =
Drive hunt within own hunting ground	N =
Comprehensive drive hunt (only shot in own HG)	N =

Do you have serious damages in you HG?	yes<br>no

In 2009 this question was supplemented with a question on sex and age of these individuals within the total annual hunting bag.

**Hunting bag** wild boar 2009/10 shot in hunting ground (total): N= ______					found dead: N= ____	
	piglets		yearlings		adults	
	female	male	female	male	sow	boar
**Hunting bag** (without found dead)						
**Found dead** at traffic						
Out of **piglets** were shot in summer (May 1st –Sept 30th)			N= ______		❑ not specified	

From 2010 on the questions were supplemented as follows:

**Wild boar population**				
Does wild boar **occur** in your hunting ground?	❑ no	❑ yes, as ❑ resident game	❑ frequent<br>❑ rare	transient
How high do you estimate the spring stock (basic stock before birth of piglets)?			N=___	❑ not specified

In the years 2010, 2011, and 2012 some questions on hunting were in much more detail:

Your opinion matters!

Regionally high wild boar populations require regulatory measures. In order to avoid unpleasant measures threatened by the EU, we hunters must succeed in reducing the wild boar population. Therefore, your experience and opinion in dealing with wild boar are important to us in order to develop problem-solving approaches based on your opinion. For this reason, in the next few years we will be asking you about various topics concerning wild boar, which may well provoke controversial discussions.

Your answers will of course be treated strictly confidentially. Nevertheless, you have the option of leaving the further block of questions on wild boar unfilled, independently of the other questions.

I cannot/would not like to give any information on the questions concerning the opinion of wild boar ❑

When were wild boar confirmed/shot for the first time in your hunting area?						❑ „allways there“	
before 1980	❑ confirmed<br>❑ shot<br>❑ unknown	1980-1989	❑ confirmed<br>❑ shot<br>❑ unknown	1990-1999	❑ confirmed<br>❑ shot<br>❑ unknown	after 2000	❑ confirmed<br>❑ shot<br>❑ unknown

In which months do the wild boar mainly give birth in your hunting area? April | M | J | J | A | S | O | [illegible]

❑ no occurence/reproduction ❑ unknown (please tick, multiple answers possible)

How many wild boar have been shot during which kind of hunt (own hunting ground in 2009/10)?

	N		N
Hide at bait	______	Drive hunt in own hunting ground r	______
Hide at damage within fields	______	Comprehensive drive hunt (only N of shot in own HG)	______
Accidentially during hide for other game	______	Specific wild boar drives (e.g in small bushes)	______
stalking	______	During harvest (e.g corn)	______
collective hide with several hunters in parallel	______	Small game drive hunt	______

How many wild boars did you shoot in which months in your hunting area?

April	M	J	J	A	S	O	N	D	J	F	March

Do you allow shooting of wild boar on small game drives? ❑ yes ❑ only piglets ❑ only animals not leading piglets ❑ any wild boar ❑ no

❑ we do not conduct small game drives

Are drive hunts in your hunting ground	❑ conductable	❑ not practical	?
Are comprehensive drive hunts	❑ conductable	❑ not practical	?

Do you conduct common hunts on wild boar? ❑ yes ❑ no

N hunts (hunting year Jagdjahr 2011/12)	hunt number	N hunters	N beaters	N dogs
____	1			
	2			
	3			
	4			

How do you conduct common hunts? (**multiple answers possible!)** ❑ no answer

Procedure

Encircling and beating

❑ thickets
❑ reed
❑ field copses

❑ with dogs
❑ no dogs

Beating of cropfields

❑ maize
❑ intertillage (e.g. charlock, radish)
❑ sunflower
❑ others ____________________

❑ with dogs
❑ no dogs

❑ Poking (stirring hunt with low pressure, few calm beaters, dogs at heel)
❑ Drive hunt with high pressure (loud beaters, barking dogs)
❑ Rummage (only dogs roaming independently)

❑ Collective hide
❑ dawn
❑ dusk
} ❑ with stirring
❑ without stirring
❑ moonlight

Hunting party

❑ solely few friends and colleagues
❑ incl. invited guests
❑ incl. paying guests

Stands (predominantly)

❑ ground level
❑ battue stands (<1,5m)
❑ raised hide (>3m)

Allowed munition

❑ rifle, lead-free
❑ rifle, leaded
❑ shotgun slugs

Organisation

❑ solely own hunting ground
❑ comprehensive with neighbours

What other game species are hunted? ❑ red deer ❑ roe deer ❑ predatory game
❑ fallow deer ❑ mouflon ❑ small game

During these hunts wild boar is… ❑ main or target species. ❑ by-catch.

What difficulties do you encounter in conducting driven hunts? ❑ not specified

❑ no difficulties
❑ organisation of hunting event
❑ building of stands
❑ „Not worth the effort“ (low wild boar density)
❑ bad shooting opportunities (e.g. lot of understorey)
❑ too few good shooters
❑ to few good beating dogs
❑ to few good trailing dogs
❑ problems with cooling game meat
❑ marketing problems

Do you bait wild boar in you hunting ground? ❑ yes ❑ no __________ N baiting sites
Do you run diversionary feedings? ❑ yes ❑ no __________ N feeding stations

Which bait do you use mainly? (only one possibility)
❑ maize ❑ grain ❑ acorn / horse chestnut
❑ other crops ❑ others

Which additional bait do you use**?** (multiple possibilities)
❑ maize ❑ grain ❑ acorn, / horse chestnut
❑ other crops ❑ pellets ❑ anderes

How much bait do you use up per year? ________ kg

animals

Supplemental Material

Explanation of hunting methods

Table S3: Explanation of hunting methods:

• single hunt	solitary hunter
a. from a hide (raised stand)	with or without baiting (little amount of food just for baiting – not feeding)
b. hiding within fields	e.g. close to damages
c. stalking	
d. accidential	by chance during a single hunt on other game
• collective hiding	simultaneous organised single hunt from hides
• drive hunts might be conducted:	conducted with dogs and beaters to hunt on hoofed game within forest
a. in own hunting ground solely	
b. comprehensive/simultaneously in several neighbouring hunting grounds	
with different "hunting pressure":	
a. poking	only dogs beating, without beaters
b. slow beating	only beaters without dogs, slowly walking
c. heavy drive hunts	heavy beating with dogs and beaters
• selective wild boar drives	specific small drive hunts just searching for wb
a. thickets	
b. groves	
c. reed	
d. intertillages	
e. other crops	
• harvest	wild boar shot during harvest (hunters waiting at the edges outside harvested fields)
• small game drive hunts	by chance during a hunt on small game

animals

MDPI

Supplemental Material

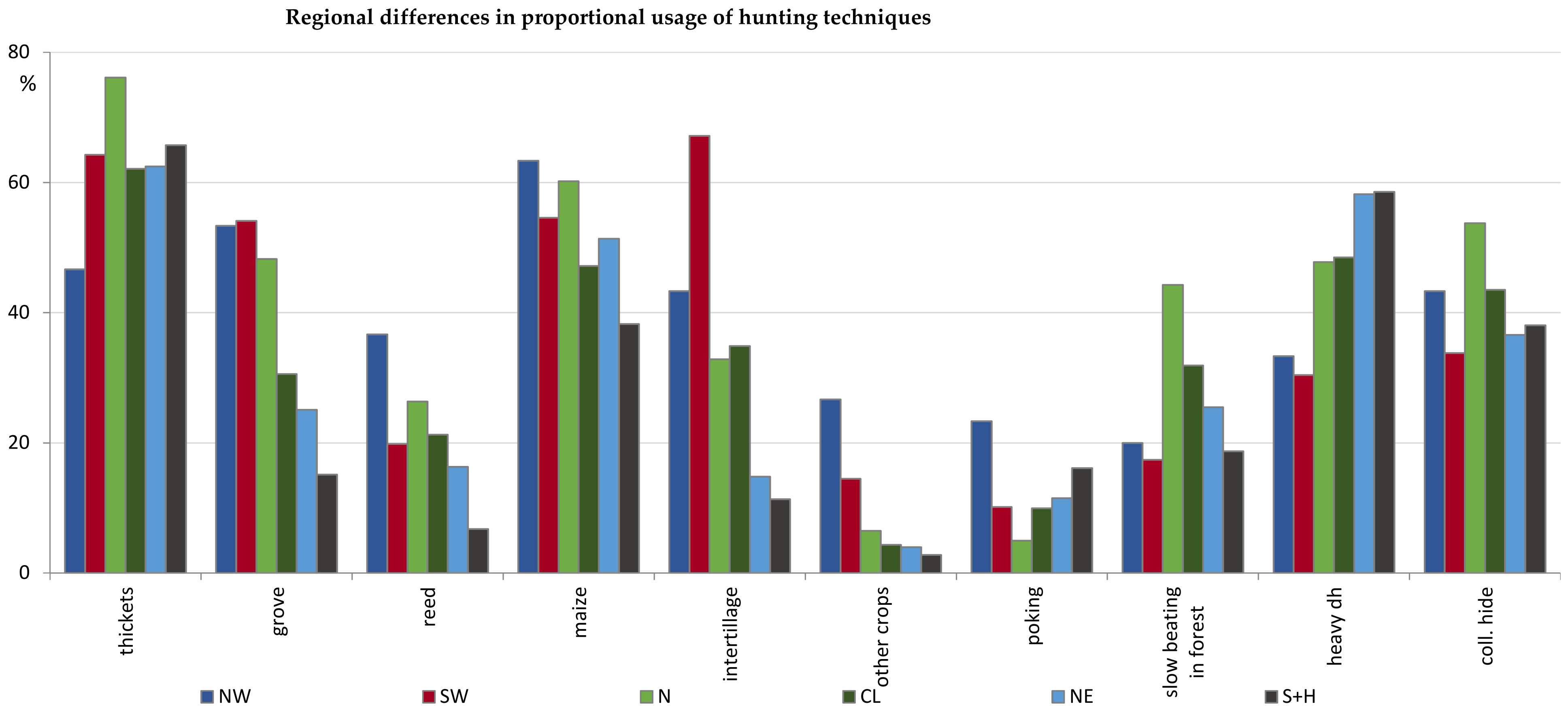

Figure S4: Percentages of hunting grounds conducting specific hunting methods on wild boar within the regions of Lower Saxony, dh = drive hunt, poking= just dogs - no beaters, N = North, S = South, W = West, E = East, CL = central Lowlands, H = Harz mountains, N total= 1,969, WTE 2012

animals MDPI

Supplemental Material S5

Development of hunting bags in German federal states

Obvious are some stagnations of the increase of hunting bags. In the 1990th classical swine fever (CSF) was roaming in Germany as well as a second epidemics of CSF in the early 2000s (Fig. SM5-1). Also after highest hunting bags in 2008/09. However, after these stagnations there was always a “jump” to new hunting bag records!

Trends for hunting bag statistics show that stagnations of the increase of hunting bags are just an intermediate pause, the trends are still exponential (Fig. SM5-2).

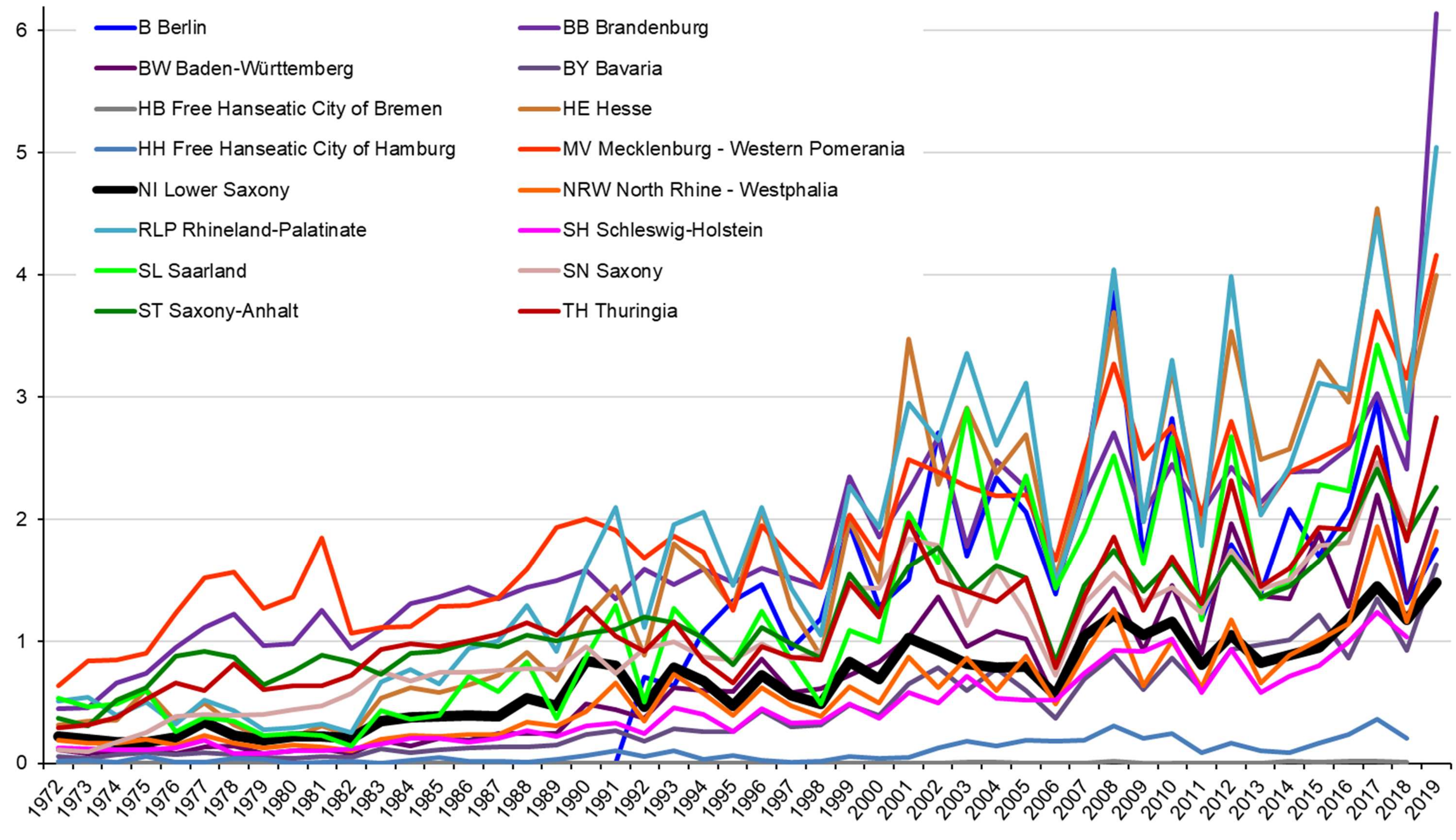

Figure S5-1: Official hunting bags statistics for the German Federal States from 1972/73 until 2019/20, expressed as HI (hunting index = shot/km²)

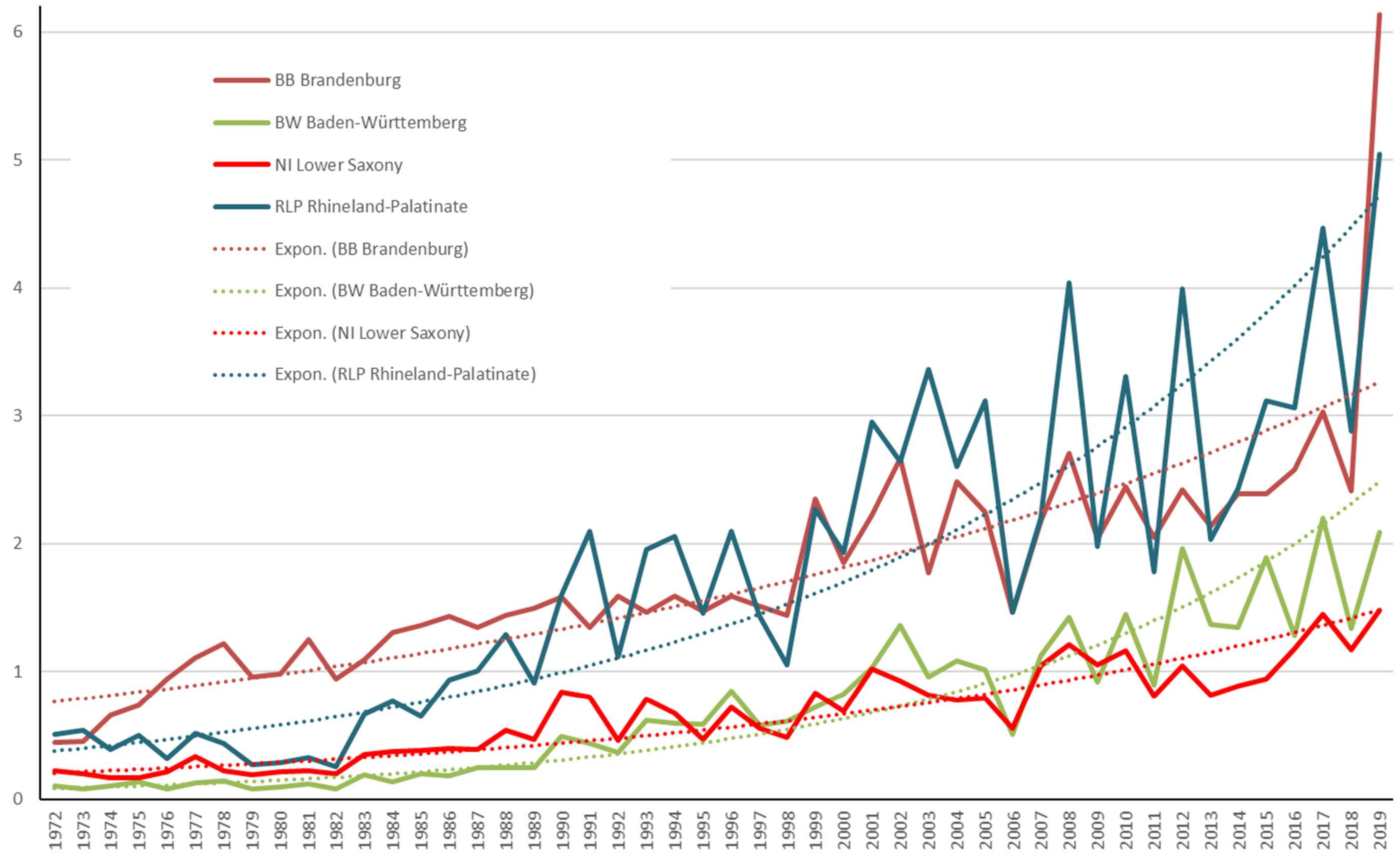

Figure S5-2: Official hunting bags statistics for the German Federal States from 1972/73 until 2019/20, expressed as HI (hunting index = shot/km²) for four federal states of Germany exemplarily.
